# Informing optimal testing and isolation strategies across different stages of the diagnostic development pipeline using mathematical models: SARS-CoV-2 in the UK as a case study

**DOI:** 10.1136/bmjph-2025-002993

**Published:** 2026-05-14

**Authors:** Ricardo Aguas, Caroline Franco, Bo Gao, Sheetal P Silal, Jared Norman, Siyu Chen, Wirichada Pan-Ngum, Susan Hopkins, Tom Fowler, Lisa White, The EY-Oxford Health Analytics Consortium, Adam Bodley, Ainura Moldokmatova, Anastasiia Polner, Angus Ferguson-Lewis, Billie Andersen-Waine Ben Lambert, Claire Keene, Emily Rowe, Gulsen Yenidogan, Kasia Stepniewska, Kweku Bimpong, Liberty Cantrell, Lisa J White, Joseph L-H Tsui, Ma'ayan Amswych, Marta Wanat, Melinda C Mills, Merryn Voysey, Muhammad Kasim, Prabin Dahal, Rachel Hounsell, Reshania Naidoo, Richard Lewis, Rima Shretta, Randolph Ngwafor Anye, Richard Creswell, Sabine Dittrich, Sarah Pinto-Duschinsky, Sassy Molyneux, Sheetal Silal, Sompob Saralamba, Sophie Dickinson, Sumali Bajaj, Sunil Pokharel, Tracy Evans, Umar Mahmood

**Affiliations:** 1 Nuffield Department of Medicine, Oxford University, Oxford, UK; 2 Biostatistics and Health Data Science, School of Medicine, Medical Sciences and Nutrition, University of Aberdeen, Aberdeen, UK; 3 Department of Statistical Sciences, University of Cape Town, Rondebosch, South Africa; 4 Department of Public and Ecosystem Health, Cornell University, Ithaca, New York, USA; 5 Mathematical and Economic Modelling, Mahidol Oxford Tropical Medicine Research Unit, Bangkok, Thailand; 6 UK Health Security Agency, London, UK; 7 Public Health Wales, Oxford, UK; 8 William Harvey Research Institute, Queen Mary University of London, London, UK; 9 Department of Biology, University of Oxford, Oxford, UK

**Keywords:** SARS-CoV-2, Epidemics, Communicable Disease Control, methods

## Abstract

**Introduction:**

Mass testing strategies recognise the importance of rapidly identifying individuals infected with potentially asymptomatic pathogens to mitigate outbreaks. A pressing question is how to design testing strategies that navigate the delicate balance between reducing morbidity and minimising societal disruption and absenteeism among key workers while balancing associated logistical and financial considerations.

**Methods:**

We use an individual-based model with explicit viral dynamics to explore the inherent trade-offs in testing regimen design and identify optimal regimens for different stages of a pandemic. Applying this framework to the SARS-CoV-2 pandemic in the UK, we evaluate the impact of testing and isolation protocols on two key objectives: protecting the vulnerable and reducing transmission. Each testing regimen is evaluated according to three outcomes: reduction in infectivity, testing efficiency and isolation efficiency.

**Results:**

Our analysis shows that these outcomes are on orthogonal optimisation axes, indicating a sharp trade-off between strategic goals. This implies that transmission reduction strategies are inherently inefficient and that regimens with high testing efficiency will always have suboptimal isolation efficiency. By focusing on testing regimens to protect vulnerable groups, we map the predicted outcomes across different stages of the diagnostic development pipeline. We also examine how adherence to testing and isolation guidelines may influence the selection of regimens aimed at reducing overall transmission.

**Conclusions:**

Based on these findings, we offer a framework to streamline the selection process for robust testing strategies spanning the duration of a pandemic, ensuring consistently high levels of adherence and thus impact.

**WHAT IS ALREADY KNOWN ON THIS TOPIC**

- Mass testing strategies could potentially have a very large impact on population level transmission of pathogens characterised by asymptomatic infectivity. In theory, if one could identify all asymptomatic cases early in their infectious period, most onwards transmission could be prevented. In practice, the effectiveness of any such strategy lies in the balance between four factors: (1) frequency of testing regardless of symptoms; (2) onset of infectivity postinfection; (3) adherence to isolation protocols and (4) duration of isolation. Ascertaining which strategy or set of strategies is optimal to control a specific outbreak is not trivial.

**WHAT THIS STUDY ADDS**

- We present a detailed sensitivity analysis that identifies the key drivers of testing strategy success, mapping the predicted outcomes of different strategies across different stages of the diagnostic developmental pipeline. We found that adherence to isolation and testing protocols is the most important driver of success for strategies aiming to control transmission through mass testing. We ultimately propose a framework that facilitates the design of robust testing strategies spanning the duration of a pandemic that ensure consistently high levels of adherence and thus impact.

**HOW THIS STUDY MIGHT AFFECT RESEARCH, PRACTICE OR POLICY**

- This study highlights how mass testing strategies that demand high levels of compliance and adherence for long periods of time are extremely fragile and can easily turn out to be suboptimal. Priority should be given to the design of more practical and consequently robust alternative strategies which could ultimately be more impactful.

## Introduction

In response to the COVID-19 pandemic, the UK government committed to mass testing, commencing in March 2020. In May 2020, the National Health Service Test and Trace was formally established, as an Executive Agency of the Department for Health and Social Care, to lead an ‘at scale’ national testing, tracing and isolating service.

Since then, several independent evaluations of the testing and tracing services delivered in England from October 2020 to March 2022 have been conducted. These evaluations were performed using mathematical models expedited to support the evidence base for testing interventions, similar to the analyses performed for other non-pharmaceutical interventions.^1–3^ All models agreed that testing and isolating only symptomatic infections was ineffective,^1 4^ since presymptomatic, or even asymptomatic individuals can potentially transmit SARS-CoV-2.^5 6^

Understanding SARS-CoV-2 dynamics within the human body is critical to the design of transmission suppression testing strategies. With sufficient knowledge of typical viral load profiles of infection, mathematical models can propose optimal testing strategies for various epidemiological contexts.^2 3 7–10^ These modelling analyses can be roughly classified into those identifying care providers (healthcare practitioners and care home workers) as the target group of the testing strategy and those investigating the deployment of mass scale testing for the entire population. Intriguingly, while the modelling methodology is similar, the outputs of interest are notably different depending on what type of testing strategy is being evaluated. When looking into testing strategies that focus on care providers, priority is put on minimising the likelihood of outbreaks and disruptions to care. In contrast, population-wide testing strategies focus shifts to transmission suppression.

Testing strategies have the potential to mitigate outbreaks by affecting how individuals spread infection if they know their infection status. A first-principles approach, avoiding assumptions about the complexities of evolving human contact networks during an outbreak, involves simulating detailed decision-making processes related to testing adherence and isolation and extrapolating the effects of these individual decisions on the potential for subsequent viral transmission. The optimal strategy for minimising SARS-CoV-2 infections encompasses the early identification and effective isolation of infectious individuals. Given an available diagnostic tool, the potential impact of any such strategy lies in the balance between four factors: (1) frequency of testing regardless of symptoms; (2) onset of infectivity postinfection; (3) adherence to isolation protocols and (4) duration of isolation. Each individual testing positive early in their infectious period and appropriately isolating until they are no longer infectious will curtail their infectivity to others. At the population level, the optimal testing and isolation strategy is then that which reduces total individual infectivity the most. Here, we outline a simulation framework that incorporates behavioural factors, within-host viral dynamics, and implementation constraints to produce key outcome evaluation metrics of testing strategy impact like population level reduction in infectivity, numbers of tests per person per month and isolation days per person per month. Our analytical approach unifies those metrics across contexts and provides a framing for the potential societal cost and logistical feasibility of any testing strategy. Importantly, a first-principles approach relies solely on our understanding of within-host viral dynamics and human adherence to policy, thus avoiding the need to make any assumptions about the underlying dynamical processes of viral transmission, which are fraught with uncertainty, extremely time-varying, and confounded by other interventions.

The ongoing challenge is to optimise testing regimens for multiple outcomes (eg, reducing transmission while minimising isolation days) under time-varying restrictions (eg, limits on the availability of rapid tests in the earlier stages of a pandemic). Here, we present a general framework that allows for scoping of any combination of pandemic scenario and testing regimens, while keeping track of the trade-offs between competing outcomes and constraints (online supplemental box 1). We apply that framework to the SARS-CoV-2 pandemic and explore the potential impact of testing and isolation protocols aimed at reaching two aims: protecting the vulnerable and reducing transmission.

10.1136/bmjph-2025-002993.supp1 Supplementary data

## Materials and methods

### Testing strategies

We developed an individual-based framework that allows us to simulate any testing strategy. We assume that testing of symptomatic individuals is a key irremovable feature of any respiratory infection control strategy. In the context of the SARS-CoV-2 pandemic, we explore the deployment of testing strategies that aim to identify asymptomatic infections given their contribution to the force of infection (FOI).^11^ We focus on two key strategies with different aims, one designed to protect those at highest risk (aim 1), another designed to reduce transmission in the whole population (aim 2). For more details on the model, please refer to the online supplemental text.

Within each simulated testing strategy, testing efforts can have two purposes:

- Regular testing to identify infections—this will also be referred to as ‘routine testing’.
- Testing to meet isolation release criteria—referred to as ‘test to release’.

Hence, for a given routine testing policy, individuals will be tested with a regularity defined by the testing frequency; those returning a positive test will trigger isolation which can take one of three forms:

- Isolate for a fixed number of days.
- Isolate and test daily until a given number of consecutive negative test results is returned.
- Isolate and follow a predetermined testing schedule with release after the first negative test.

We also incorporate two different testing methods in the model:

- rapid tests, with instantaneous turnaround time (eg, lateral flow diagnostic tests (LFDs)).
- PCR, with a fixed turnaround time.

The two diagnostic methods are parametrised with different specificity, sensitivity, and limit of detection, based on previous comparative evaluations for COVID-19 testing.^12^ We account for how test sensitivity varies as a function of the individual viral load, according to data in Pickering *et al*,^12^ which is why test sensitivity is given as a range rather than a single value in online supplemental table S1.

Due to its individual-based nature, the model can easily be applied to specific age subgroups and allows for the two testing methods to be used simultaneously. The proportion of individuals using each testing method can be set at different levels for both routine testing and test to release protocols. We should note that PCR is only implemented as a viable routine test for aim 1, where testing is restricted to those at highest risk. For aim 2, reflecting testing policies for the wider population, routine testing was simulated exclusively with rapid LFD.

Behavioural aspects were also considered through the introduction of different sources of poor adherence:

- Adherence to routine testing guidance, translating into a proportion of expected tests missed in a 30-day period.
- Adherence to testing when isolating, i.e. the proportion of missed scheduled tests while isolating.
- Adherence to isolation protocol guidance, which is a summary measure of the reduction in total contacts when isolating.
- Non-consent to testing guidance, reflecting the proportion of people in the target population who refuse to have any test.

When designing a testing strategy, the key operational parameters to optimise are the routine testing frequency, the choice of diagnostic method(s) and the isolation protocol (guidance on how to reduce numbers of contacts and the testing schedule while isolating).

### Viral load profiles

To inform individual infectivity profiles, we used the findings in Jones *et al* 13 to parametrise a piecewise viral load function which depends on age and clinical outcome, as well as individual variability covariates. The same study^13^ estimated a time lag of 4.3 days between the onset of viral shedding and peak viral load and found an almost linear relationship between age and the peak viral load for all symptomatic presentations (presymptomatic, asymptomatic and mildly symptomatic (PAMS) and hospitalised cases). Following cell culture experimental results,^13^ we modelled a sinusoidal relationship between viral load and culture probability, the latter a proxy for viral infectivity.

We thus model individual viral load as a function of time, as a piecewise linear function, following:

(1) $$log_{10}(V)=\left\{ \begin{array}{c} log_{10}\left(V_{max}\right)\frac{t}{t_{max}},t\leq t_{max}\\ log_{10}\left(V_{max}\right)+r(t)\left(t_{max}-t\right),t§gt;t_{max} \end{array}\right.$$

where t is the time in days since exposure and *t_max_* is the day in which peak viral load is reached. This piecewise representation of viral load profiles has been further validated by other studies establishing a logarithmic relationship between viral load and PCR cycle threshold (Ct) values.^14 15^ Understanding the relationship between Ct values and viral load is essential for establishing detection thresholds and comparing diagnostic tests across manufacturers. In our framework, we specify sensitivity and specificity values for each log 10 viral titre value.

To represent the active viral load profile of each individual, we introduce a decay rate, r(t) :

(2) $$r(t)=r_{0}\left(\frac{a+t}{\alpha +t_{\max}}\right)$$

where a is adjusted to represent a faster than linear decay rate in viral load after the peak. This adjustment prevents overestimating the amount of viable viral particles with infective potential at the end of the infection. Active viral load profile can be used to produce infectivity profiles that are directly comparable to culture probability empirical data. We calibrated a generalised logistic regression to the sigmoidal relationship between viral load and infectivity (online supplemental figure S1C) observed in^13^ which can be phenomenologically described by:

(3) $$P\left(log_{10}V\right)=\frac{0.958}{1+5^{\left(7.12-log_{10}V\right)\times 0.85}}$$

Using equations (1) to (3), we can obtain individual infectivity and viral load profiles, such as the ones shown in online supplemental figure S1A,B. These illustrate the difference in model-generated profiles with and without considering the nonlinear decay rate of viral activity after the peak. Clearly, discounting inactive copies of viral RNA (that are not viable for onwards transmission) could have a large impact on resulting infectivity timelines after the viral load peak.

### Age and clinical outcome dependence

Based on Jones *et al*’s results,^13^ we were able to extrapolate a linear relationship between age in years (x) and peak viral load difference (*Δ V*), for a fixed clinical outcome. This relationship can be written (approximately) as:

(4) $$\Delta \log_{10}\left(\hat{V_{\max}}(x)\right)=0.012x$$

As V and $V_{max}$ also depend on each individual’s clinical outcome (y), we introduce a scale parameter, $k_{y}$ , to account for differences between hospitalised, PAMS (presymptomatic, asymptomatic and mildly symptomatic) and other groups. Hence, peak viral load can be written as

(5) $$V_{\text{max }}(x,y)=\hat{V_{\text{max }}}(0)10^{0.012x+k_{y}}$$

with $k_{y}$ being 1 for hospitalised, 0.25 for PAMS and 0.35 for other groups.

In addition to being dependent on age and outcome, viral load profiles and their corresponding infectivity profiles are also assumed to vary across individuals within the same group. This is accounted for by drawing parameter values from appropriate distributions, rather than assuming fixed values. Details about the distributions used are provided in online supplemental table S1, and the profiles generated using the above framework for a set of 10 000 individuals are depicted in online supplemental figure S2. A reconciliatory breakdown of the distribution of clinical outcomes across age groups is shown in online supplemental figure S3.

### Evaluation metrics

The integral of the infectivity (equation (3)) curve over time can be interpreted as the aggregate potential for onward transmission over a certain period, ${I\;|\;}_{t_{2}}^{t_{1}}$ which can be calculated as:

(6) $$I{|}_{t_{2}}^{t_{1}}=\int_{t_{1}}^{t_{2}}\left(\left\{ \begin{array}{ll} \frac{0.958}{1+5^{\left(7.12-\log_{10}(V_{\max})\frac{t}{t_{\max}}\right)\times 0.85}} & t\leq t_{\max}\\ \frac{0.958}{1+5^{\left(7.12-\log_{10}(V_{\max})+r_{0}\left(\frac{a+t}{a+t_{\max}}\right)(t_{\max}-t)\right)\times 0.85}} & t§gt;t_{\max} \end{array}\right.\right)\;dt$$

The population-wide impact of a testing and isolation strategy on overall transmission potential can then be assessed by calculating the collective relative decrease in infectiousness, accounting for the reduced number of contacts when isolating.

(7) $$\sum {\;}^{N}I_{R}{|}_{t_{2}}^{t_{1}}=\frac{{\sum I_{\text{interv }}|}_{t_{2}}^{t_{1}}}{{\sum I_{\text{control }}|}_{t_{2}}^{t_{1}}}$$

This measure of relative infectiousness reduction (due to testing and isolation), compared with the overall infectiousness potential in a no asymptomatic isolation scenario is the main output metric and will sometimes be referred to as impact.

To translate the impact of a selected testing strategy from reduction in transmission potential to reduction in transmission (FOI), we transformed the age-structured impact output using established social contact matrices.^16 17^ Note that the relative infectiousness defined above can be calculated for any population subgroup, such as an age group. A vector of relative infectiousness reduction (by age) can be translated into FOI reduction by multiplying this vector by the relevant social contact matrix, *C*:

(8) $$\vec{PRT}d=C\vec{{I_{R}|}_{t_{2}}^{t_{1}}}$$

For each testing regimen, we thus keep track of three key evaluation metrics that allow us to frame the predicted testing strategy impact in terms of its potential societal cost as well its logistical feasibility:

- Infectivity reduction—the difference in the infectivity profile integral that serves as a proxy for transmission impact. Testing strategy evaluations have so far only focused on this aspect.
- Testing efficiency—incremental testing effectiveness ratio (ITER), reflecting the percentage infectivity reduction per test performed. This gives an idea of how many tests need to be performed to achieve a certain target impact, thus having direct implications on the financial efficiency of the strategy.
- Isolation efficiency—infectivity reduction per isolation day, the incremental isolation effectiveness ratio (ISER), accounting for the percentage infectivity reduction per isolation day. This reflects how disruptive a strategy needs to be to achieve a certain target impact, thus offering some perspective into the proportionality of the expected societal impacts given the predicted number of isolation days required.

The objective of the analyses described here is to learn from the recent pandemic experience and inform future contingency planning around, for instance, the optimal use of tests when test availability is increasing over time or when different strategies need to be deployed for different subpopulations (online supplemental box 1).

To evaluate aim 1, we explored which strategies can provide maximum impact (maximise infectivity reduction) while minimising absenteeism (maximise ISER) among key workers, given the testing capacity at each epidemic stage. We have prioritised simplicity in guidelines when selecting strategies for each phase and predicted the testing capacity required for switching from one strategy/stage to the next (table 1).

**Table 1:** Strategies explored in aim 1

Strategy	Routine testing frequency	Routine testing diagnostic	Isolation testing frequency	Isolation testing diagnostic	Stage
RdId	daily	LFD	daily	LFD	3
RdI7pcr	daily	LFD	weekly	PCR	3
RdI7lfd	daily	LFD	weekly	LFD	3
RdI14	daily	LFD	none	none	3
R3wId	3× per week	LFD	daily	LFD	3
R3wI7pcr	3× per week	LFD	weekly	PCR	3
R3wI7lfd	3× per week	LFD	weekly	LFD	3
R3wI14	3× per week	LFD	none	none	2
RwId	weekly	PCR	daily	LFD	3
RwI7pcr	weekly	PCR	weekly	PCR	1
RwI7lfd	weekly	PCR	weekly	LFD	2
RwI14	weekly	PCR	none	none	1
RbwId	biweekly	PCR	daily	LFD	3
RbwI7pcr	biweekly	PCR	weekly	PCR	1
RbwI7lfd	biweekly	PCR	weekly	LFD	2
RbwI14	biweekly	PCR	none	none	1

LFD, lateral flow diagnostic.

For aim 2, we explored several options for testing schedules and isolation (fixed period, test to release) for a range of levels of consent to testing and adherence to testing and isolation. Note that the large sensitivity analysis shown here contains simulations for all combinations of parameter values in table 2.

**Table 2:** Parameters explored in aim 2 analyses

Parameter	Values/options considered
Behavioural	
Isolation testing adherence	{100%, 75%, 50%}
Isolation contact reduction	{100%, 80%, 60%}
Routine testing adherence	{100%, 75%, 50%}
Non-consent to testing	{20%, 10%, 0%}
Epidemiological	
Prevalence	{10%, 5%, 1%}
Operational	
Routine testing interval (days)	{7, 2, 1}
Isolation protocol	Fixed isolation period (days): {14, 7} Consecutive negative tests to release if testing daily: {2, 1} Testing interval with release on first negative test (days): {5, 2}

This aim can only be pursed during stage 3 of the pandemic (online supplemental box 1) and, in this exploratory analysis, we simulated all tests to be LFDs.

LFD, lateral flow diagnostic.

### Multivariate analysis

To aid interpretation of the results, a principal component analysis (PCA) was employed to visualise the relationship between the simulated output metrics: infectivity reduction, testing efficiency and isolation efficiency. The resulting loading vectors reveal the directionality of the metric embedding on the new coordinate system, indicating where strategies with a high infectivity reduction outcome, for example, are expected to fall in the transformed coordinate plot.

We performed a k-means clustering analysis on the strategy coordinates across the first two principal components to infer if/how the testing strategies cluster into distinct groups. To understand whether the resulting attribution of group membership is meaningful, we reverse-engineer the dimension reduction process to see how the different strategies scatter in the planes defined by combinations of our output metrics.

## Results

**Figure 1:**
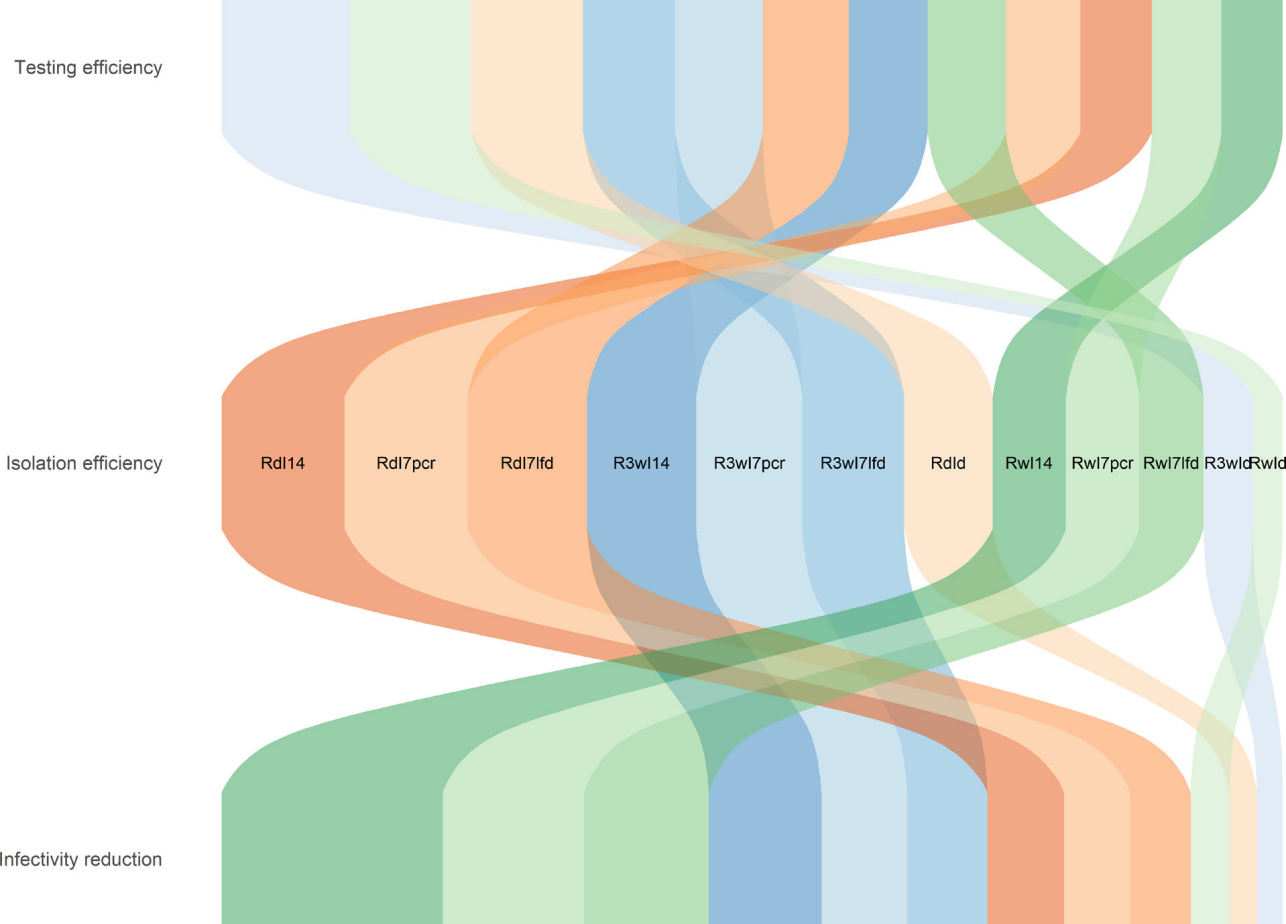
Alluvial plot displaying the ranking of each aim 1 intervention for each outcome metric. Top ranking strategies for each metric are situated to the left of the plot. Note that the within output metric values have been standardised to make the width of each ribbon convey how it compares against the other strategies for a given output. The wider the ribbon, the more dominant the strategy, that is, it achieves an output value that is much larger than the mean value across all strategies. The colours of the ribbons are linked to the routine testing frequency, with green representing weekly, blue representing three times per week and orange representing daily testing. Strategies are fully described in table 1.

Results from our simulations reveal that testing strategies focusing on key workers can have a significant impact on SARS-CoV-2 transmission. Figure 1 illustrates how the strategies explored in Aim 1 (table 1) rank according to their expected outcome metrics. It reveals strict trade-offs, with no strategy being predicted to achieve good outcomes for all three metrics. There is a noticeable variation in testing efficiency among strategies, with the ribbons crossing the vertical centre line as they descend the plot. This indicates that strategies maximising testing efficiency tend to result in lower impact and low isolation efficiency. All strategies appear to have similar isolation efficiency, which signals that the impact of key worker testing strategies is more sensitive to changes in testing implementation details rather than the isolation protocol itself. This is reinforced by the predominance of high testing frequency (daily and three-times per week) strategies to the left of the infectivity reduction plane (the orange and blue ribbons, respectively), that is, the highest impact strategies.

**Figure 2:**
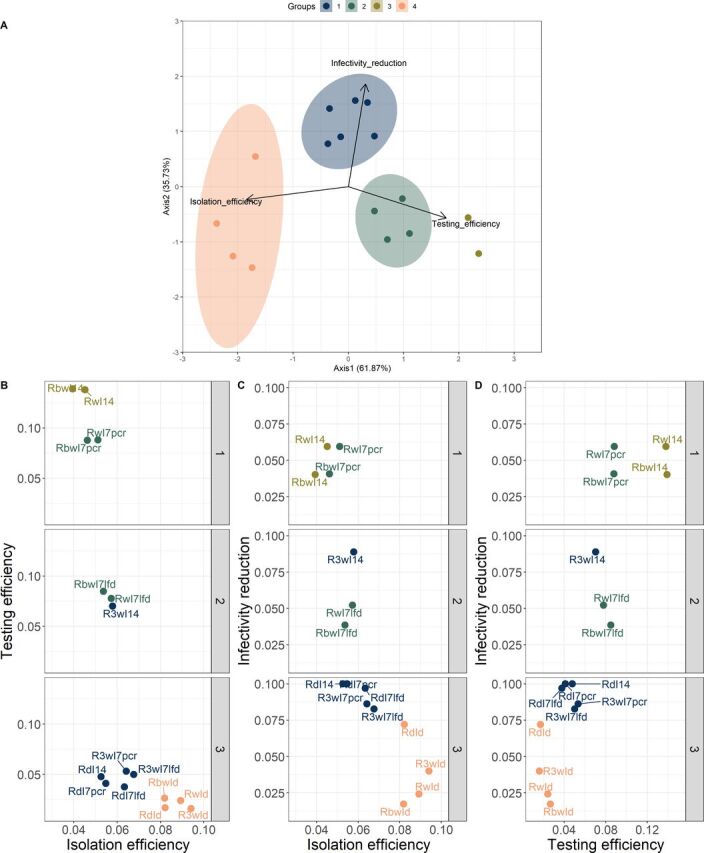
Testing strategy outcome trade-offs. (A) First and second principal components extracted from the aim 1 simulation set, with group membership assigned through K-means clustering. (B–D) Testing strategies for key workers across planes defined by combinations of testing efficiency, isolation efficiency and infectivity reduction for each stage of a pandemic (given by each of the rows), as defined in table 1.

Interestingly, within the orange shaded ribbons (highest impact), the worst performing strategy includes daily testing while isolating, suggesting that the poor sensitivity of LFD tests can be responsible for a premature release from isolation if implementing very regular testing isolation protocols. The trade-offs identified in figure 1 are confirmed by performing a PCA on the set of outcome metrics for each simulated strategy. The loading vectors plotted on the first two principal components plane are nearly orthogonal, strongly indicating that simultaneous optimisation across all dimensions is impossible (figure 2A). Strategies cluster into four groups (online supplemental figure S4) that can be easily labelled (figure 2B) after reverse-engineering the dimension reduction process:

- Group 1 (navy)—high impact, low testing efficiency, average isolation efficiency.
- Group 2 (dark green)—average across all three metrics.
- Group 3 (lime green)—high testing efficiency, low isolation efficiency, average impact.
- Group 4 (salmon)—high isolation efficiency, low testing efficiency, average impact.

Figure 2B indicates that strategies which maximise one of the desirable Aim 1 features (maximal isolation efficiency and thus lower rates of care disruption due to absenteeism) are not particularly impactful. More importantly, they are only possible to deploy during the later stages of a pandemic, once testing capacity has increased considerably and daily testing becomes possible. The most impactful strategies lie in the middle of the isolation efficiency axis but sit on the lower end of both isolation and testing efficiencies. Figure 2C,D illustrates that strategies incorporating more frequent routine testing have larger potential impact, but reduced testing efficiency. As noted earlier, daily testing options for test to release protocols yield the worst infectivity reduction results due to issues with test sensitivity. A general mapping of these results suggests that adopting weekly testing regimens (weekly routine testing and weekly testing while isolating) is optimal in the early course of a pandemic. A change in strategy is only warranted once the testing capacity is sufficient to allow for daily routine testing of the population subgroups of interest. Test to release protocols should be carefully tailored to the test’s ability in preventing false negative results (sensitivity). Examining the impact of each strategy on the FOI experienced by different age groups offers further evidence for selecting the testing strategy best suited for a given aim (online supplemental figure S5). It should be noted that we have not explored adherence/compliance issues in the aim 1 analysis. An extensive sensitivity analysis of human behavioural determinants of testing strategy success was performed for aim 2, and the results obtained there should be directly translatable to aim 1.

Our extensive model sensitivity analysis for mass-scale testing strategies (aim 2) reveals that parameters related to isolation guidelines have a disproportionate impact on isolation efficiency and infectivity reduction, while routine testing frequency is the main determinant of testing efficiency (online supplemental figure S6). It is important to note that all output metrics are insensitive to prevalence, which is reflective of how the model is set up. Given we do not model transmission dynamics, we do not take into account the non-linear effects that are intrinsic to such systems. Instead, we retrieve a decrease in infectivity, which is almost linearly dependent on prevalence, thus generating a similar percentage reduction across prevalence levels.

**Figure 3:**
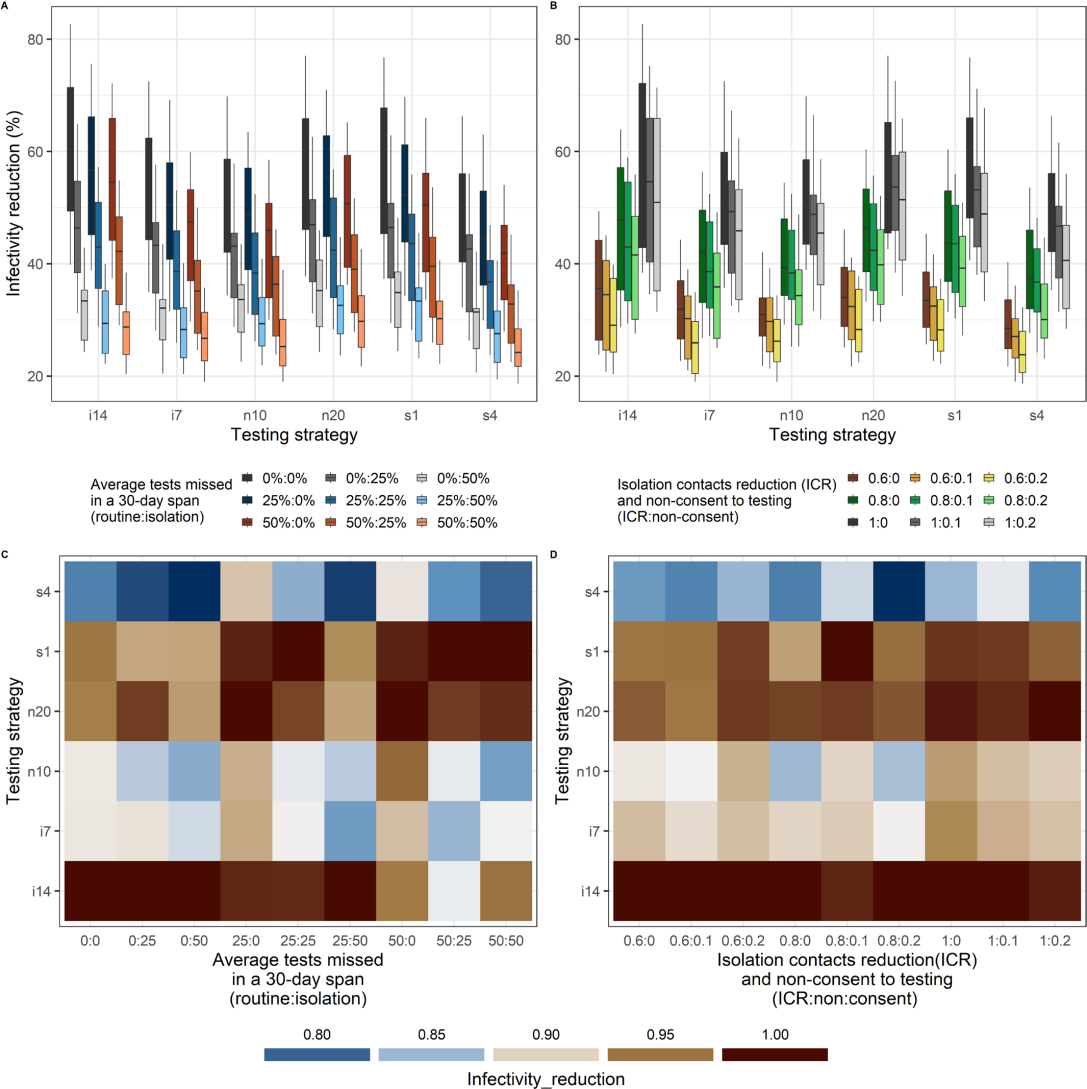
Infectivity reduction dependence on mass-scale testing adherence (left), consent and reduction of contacts while isolating (right). These simulations assumed 1% prevalence and a daily routine testing regimen. Panels A and B summarize infectivity reduction across the explored parameter sets, with boxes displaying interquartile (IQR) range and whiskers extending to 1.5 times the IQR. Panels C and D re-orient the results in panels A and B to show which strategies have the highest impact for each set of adherence and consent. Testing strategies are coded as follows: i14 – fixed 14 day isolation period; i7 – fixed 7 day isolation period; n10 – isolation release on first negative test when testing daily while isolating; n20 – isolation release on second consecutive negative test when testing daily while isolating; s1 – testing every two days with release on first negative test while isolating; s4 – testing every 5 days with release on first negative test while isolating.

To better inform testing policy design, we performed a full multivariate sensitivity analysis to uncover potential implementation efficiency barriers. Figure 3 illustrates how predicted infectivity reduction depends on 4 of the most determinant model parameters as identified in online supplemental figure S6. There is a stark visual difference across changes in isolation contact reduction, ICR (different colours in figure 3B), and isolation testing adherence values (shades of the same colour in figure 3A). There is little difference across the explored testing consent (shades of the same colour in figure 3B) and routine testing adherence (colours of the same shading in figure 3A). How these results hinge on routine testing frequency is explored in online supplemental figure S7. For a daily routine testing frequency, isolation test adherence and reduction of contacts while isolating have a significant effect on the level of infectivity reduction that can be achieved. Increasing the time gap between routine tests would lower the potential impact of the strategy (x-axis). A well adhered to weekly testing policy and a poor adherence daily testing policy have almost precisely the same predicted impact. Again, non-consent to testing has a negligible effect size.

## Discussion

It is important to conceptually understand what effective testing strategies can have success at mitigating the impact of outbreaks for pathogens with asymptomatic transmission. If done daily, mass-scale testing could prevent over 90% of the transmission potential of asymptomatic individuals and about a third of the presymptomatic transmission potential of individuals who will later develop symptoms (online supplemental figure S8). Understandably, logistical practicalities and their volatility over time, and individual adherence and compliance to testing and isolation protocols emerge as the limiting factors to such strategies. Clear and straightforward protocols, such as testing if symptomatic and isolating for a fixed period, generally result in higher adherence^18^ and are simpler to implement.^19^

Here, we look at testing policy performance on three different axes and explore which testing and isolation protocols might be preferable at different diagnostic development stages. We purposefully compare strategies trying to maximise different metrics under different constraints (online supplemental box 1). Whereas aim 2 is only constrained by the human behavioural element of the strategy, Aim 1 involves a series of operational and logistical constraints, such as ensuring quality of care and limitations in testing capacity. The analyses described here aim to learn from the recent SARS-CoV-2 pandemic and inform future contingency planning around, for instance, the optimal use of tests when test availability is increasing over time or when different strategies need to be deployed for different subpopulations. For both aims, we evaluate the reduction in population FOI incurred by the reduction in population infectiousness through isolation. In essence, we summarise the decrease in potential onwards transmission of SARS-CoV-2 given a pre-set prevalence level. Consistent with the literature^20^ we find that testing strategies in healthcare settings and care homes could have significant potential to reduce overall transmission potential, favouring regular rapid testing over PCR testing^20–23^ when such capacity exists.

Secondary outcomes, namely the ITER and average number of isolation days per person per month (a proxy for societal cost of isolation). We thus go beyond typical analyses that evaluate testing strategies in terms of a single primary outcome—a measure of morbidity or mortality—and investigate which operational parameters are key drivers of each of the outcomes mentioned above. We add behavioural factors to the logistical and operational considerations of testing regimens to purposefully allow comparison of more demanding strategies (very frequent testing and complex test to release isolation protocols) against simple to follow and less demanding strategies.

We predict that population adherence to both routine testing and testing while isolating is the most influential factor on infectivity reduction and outweighs the choice of regimen (figure 3). Also, as adherence decreases, the rankings of predicted optimal regimens are not preserved. We conclude, therefore, that if infectivity reduction is the only consideration, it is best to select the regimen that the population is most likely to adhere to, which is a choice to be guided by behavioural research rather than modelling results. However, if testing or isolation efficiency is an important consideration, an analytical approach to the choice of regimen becomes crucial.

Our results indicate that it is not possible to select a single testing regimen for a new pandemic disease that will cover every stage of the pandemic or every use case for key subpopulations. We have shown that the various options for regimens form clusters with the following characteristics:

- High impact, average isolation efficiency, low testing efficiency.
- Average impact, high isolation efficiency, low testing efficiency.
- Average impact, low isolation efficiency, high testing efficiency.
- Average impact, average isolation efficiency, average testing efficiency.

Designing health policy is highly complex, requiring the alignment of societal, logistical, financial and epidemiological factors. In the context of the COVID-19 pandemic, the very first testing policy was drafted when testing capacity was limited, thus constraining their implementation and mandating prioritisation. It also relied on high population adherence in response to an emerging threat. We contend that expectations of population adherence should be tailored to logistical considerations, particularly as policies evolve as a response to the epidemiological context and increased testing capacity. Modelling can help determine which strategies are likely to produce the most impact with optimal adherence. However, it cannot predict the expected adherence following the implementation of any testing policy. Given how sensitive our estimates of impact are to testing adherence, we recommend that a collection of regimens with behavioural similarities should be initially derived and deployed in sequence as the pandemic unfolds. Those regimens could then be assigned to different subgroups at different stages or when appropriate, with transitions being more easily communicated at logistically feasible timepoints defined by modelling and measured using routine data. We define such a collection of regimens as an adaptive strategy. Adaptive strategies should adapt as the outbreak evolves and logistical constraints are alleviated but also be reactive to real-time testing adherence, which should be continually monitored. Low levels of adherence might warrant proposal of a revised adaptive strategy.

We propose that a way to design an adaptive strategy is to first evaluate which strategic options best balance all desirable outcome metrics and from those select a regimen that is simple to adhere to from a behavioural perspective. The next step would be to explore the clusters of regimens that align more strongly with each metric and select regimens that are behaviourally similar to the first selection. An example of such an adaptive strategy design is the collection: RwI7pcr, RwI7lfd, RdId. It is important to note that each regimen employs a single type of test and maintains the same testing frequency both in and out of isolation, thereby reducing implementation barriers. The weekly PCR regimen, RwI7pcr, is optimised for testing efficiency, whereas the daily Rapid Diagnostic Test (RDT) regimen, RdId, is optimised for infectivity reduction and isolation efficiency. The weekly RDT regimen, RwI7lfd, is a useful intermediate step while RDT availability is being scaled up and/or testing is extending to more subgroups. Other adaptive strategy designs could focus on similarities in the duration of isolation or a fixed frequency of testing to find suitable collections.

In conclusion, we present a modelling approach that can be used to inform testing response to a new pandemic challenge. We show how a modelling approach may be used to scope the options for testing regimens and explore how they may combine to form adaptive strategies while taking unavoidable trade-offs into account. We also provide guidance on the relevance of reaching high levels of testing adherence for the strategic aims to be achieved and stress the importance of being vigilant to ensure that if adherence drops to low levels, a revised adaptive strategy including prioritisation of compliance in high-risk groups should be considered. We also stress that it is impossible to optimise a testing strategy along all outcome axes: impact on transmission, testing efficiency (financial cost balancing) and isolation efficiency (societal impact balancing). Testing policy design will always be fraught with ethical conundrums and the inevitable trade-offs laid out herein must be navigated for policy makers to align their ethical compass with achievable epidemiological outcomes. Ultimately, strategies could be designed to maximise two of those metrics simultaneously, but not all three.

### Limitations

We calibrated our model to empirical data from a particular study^13^ which can raise some concerns about the generalisability of our findings. We note that corroborating studies have since been published such as a study by Stankiewicz Karita *et al* 24 looking at the association between symptom onset, disease severity and viral load profiles via a piecewise, linear mixed-effects model. They estimated similar viral load kinetics and identical timing of symptom presentations relative to peak viral load. We should note that as the pandemic progresses and the pathogen continually adapts to the human host, changes to the typical viral load kinetics are expected to take place.^25^ As such, our understanding of the impact of asymptomatic isolation on population level transmission should be constantly updated as the contribution of asymptomatic infections to transmission changes.

To ensure that our model is realistic, we compared our model’s predictions against the results of the singular rapid community testing synthetic control study conducted in Liverpool from November 2020 to January 2021.^17 18^ That open access antigen self-testing strategy included guidance to isolate for 10 days following a positive result and a ‘test-to-release’ option for contacts of confirmed infected individuals. We adjusted our framework to mimic that strategy by simulating two regimens: (1) routine testing followed by a fixed period of isolation of 10 days if positive and (2) routine testing followed by daily testing while isolating with release on first negative result. The remaining operational parameters were informed by the population behaviour reported in the study:

- ‘Between 6 November 2020 and 30 April 2021, 283 338 (57%) Liverpool residents took a test using the Innova SARS-CoV-2 antigen rapid antigen lateral flow device (LFD).’^17^
- ‘Twice weekly testing was the target. Between 6 November 2020 and 30 April 2021, 290 161 residents had an LFT test. Of these 54% did not return for a second test (as of 30 April 2021), while 16%, 27% and 33% returned for another test within 7 days, 14 days and 28 days of their first test, respectively.’^17^

This translates roughly to the following simulation settings: proportion of the population who will not participate in routine testing of 0.43; a testing interval of 28 days; and 6 routine testing days missed out of 10. The reported impact of 21% (12% to 27%) reduction in cases up to mid-December^17^ can serve as a framing reference for our model. Assuming a prevalence of 2%, based on the national prevalence at the time,^20^ we predict reductions in onwards transmission of around 16% to 20% (online supplemental figure S9) for the two preset regimens described above.

We use a conservative assumption that there is no relevant herd immunity effect within the populations/settings modelled. In this way, we can interpret the impact of our results (reduction in infectivity and FOI directly as impact on hospitalisations and deaths (accounting for those indirect effects would result in higher reduction in hospitalisations and deaths than predicted here). Given that we do not model transmission dynamics, we do not take into account the non-linear effects which are intrinsic to such systems. This could be relevant when simulating testing regimens on small subgroups/networks of people. We also do not explore the value of pooled sampling which has shown to be potentially efficacious elsewhere.^7 26 27^

## Data Availability

All data relevant to the study are included in the article or uploaded as supplementary information. All code used to perform the analyses and a link to the app are available at https://github.com/ricardoaguas/test_and_trace.
