# Supplementary material for: Informing optimal testing and isolation strategies across different stages of the diagnostic development pipeline using mathematical models: SARS-CoV-2 in the UK as a case study
Source: BMJ Public Health. 2026 May 14;4(2):e002993. doi: 10.1136/bmjph-2025-002993 (PMC13202186; doi:10.1136/bmjph-2025-002993)
Supplement: Supplementary data [file bmjph-4-2-s001.pdf]

Supplementary Box 1. Foundational aspects of the aims explored and characterisation of pandemic stages.

Aim 1

Protecting the vulnerable during different stages of the diagnostic development pipeline

- Objective is to streamline testing regimens for a population of key workers across different stages of the epidemic (below), characterised by varying testing capacity.
- High isolation efficiency ensures quality of care, aiming to provide maximum protection while minimising absenteeism.
- Simplicity is key when designing strategy guidelines for each phase.
- Strategy must adapt to the change in testing capacity/epidemic stage.
- Focus is on reducing mortality.

Aim 2

Reducing transmission while minimising isolation and accounting for adherence

- Objective is to streamline testing regimens to account for poor adherence to complex guidelines.
- Choice of testing (symptoms trigger isolation; routine testing regardless of symptoms) and isolation (fixed period, test to release) is dependent on levels of consent to the intervention, compliance with testing, and adherence to isolation.
- Simplicity and consistency are key to ensure population adherence.
- Priority is to maximise impact while maintaining high testing efficiency.
- Focus is on reducing transmission.

Stage 1

Rapid tests not available

Limited PCR capacity

Optimise for testing efficiency

Stage 2

Limited PCR and LFD capacity

Optimise for testing efficiency

Stage 3

PCR and LFDs available at scale

Optimise for isolation efficiency

Table S1: Model parameters.

Parameter	Definition	Value/central estimate	Unit	Notes	Source
Infection					
$\widehat{V_{max}}(0)$	Maximum viral load at age 0	$10^{7.2}$	viral copies	Normal distribution (mean = $10^{7.2}$, SD = 0.151)	Calibrated to profiles in [1]
t_{max}	Time from infection to peak vial load	4.2	days	Normal distribution (mean = 4.2, SD = 0.113)	Calibrated to profiles in [1]
r	Rate of decline of viral load after peak	0.17	-	Normal distribution (mean = 0.17, SD = 0.002)	Calibrated to profiles in [1]
days_symptoms_shape, days_symptoms_scale	Time of symptom onset	~ 6.7	days	Gamma distribution (shape = 22.81, scale = 0.25)	Calibrated to profiles in [1]
α	Active virus decay rate	100	-	-	Calibrated to profiles in [1]
k_y	Outcome scale factor	1 for hospitalised, 0.25 for PAMS and 0.35 for other groups	-	-	Calibrated to profiles in [1]
prob_symptoms_other	Probability of symptom development in "others" group	25	%		Assumed
Diagnostic tools					
LOD	Rapid test viral limit for detection	10^6	viral copies	Disease dependent	[2]
specificity	Rapid test specificity	99	%	Disease dependent	[2]
paraml_tdiag_sensitivity["rapid"]	Rapid test sensitivity	(65, 99)	%	The value varies in the stated range with viral load level (above the LOD)	[2,3]
lfd_turnaround_days	Rapid test turnaround time	0	days	Turnaround time is assumed negligible	Assumed
Pcr_LOD	PCR viral limit for detection	10^4	viral copies	Disease dependent	[2]
Pcr_specificity	PCR specificity	99	%	Disease dependent	[2]
paraml_tdiag_sensitivity["pcr"]	PCR sensitivity	(0.65, 0.99)	%	The value varies in the stated range with viral load level (above the LOD)	[2,3]
pcr_turnaround_days	PCR turnaround time	2	days	-	
Interventions					
test_interv	Target interval between tests (for all outcomes)	(5,30)		Range presented in contour plots/policy choice	-
test_day_active	Time between symptom onset and test (only of symptomatic outcomes)	~ 1	days	Policy choice/Gamma distribution (scale = 1, shape = 1) truncated at the symptom start date	-

param_tdiag_use_pcr_prob [“regular”]	Proportion of PCR tests performed during regular testing	0-100	%	Policy choice	-
param_tdiag_use_pcr_prob [“isolation”]	Proportion of PCR tests performed during testing to release	0-100	%	Policy choice	-
paraml_v2_ttr	Number of negative test results to release	1 or 2	-	Policy choice (daily test to release)	-
paraml_inter_scheduled_release_times	Test to release schedule (test to release)	(2,40)	days	Policy choice (scheduled test to release): a different schedule (vector of days) is set up for each strategy	-
non_compliance_rate_test	Non-compliance with testing	10	%	Behaviour assumed consistent throughout all considered groups/Beta distribution (shape1 = 0, shape2 = 10)	Assumed
non_consent_prob	Non-consent to the testing programme	10	%	Behaviour assumed consistent throughout all considered groups	
adherence	Reduction of effective contacts while isolating	80	%	Behaviour assumed consistent throughout all considered groups	Assumed
Costs					
cost_rapid	Rapid test unit cost	6.06	GBP	Default value based on evidence synthesis report	[4]
cost_pcr	PCR unit cost	68.34	GBP	Default value based on evidence synthesis report	[4]
cost_pptestrapid	Rapid test cost per person	0	GBP	Default value based on evidence synthesis report	[4]
cost_pptestrapid	PCR cost per person	0	GBP	Default value based on evidence synthesis report	[4]
cost_ppiso	Cost per patient per day isolated	0	GBP	Default value based on evidence synthesis report	[4]
cost_fixedpp	Fixed costs per person tested	0	GBP	Default value based on evidence synthesis report	[4]

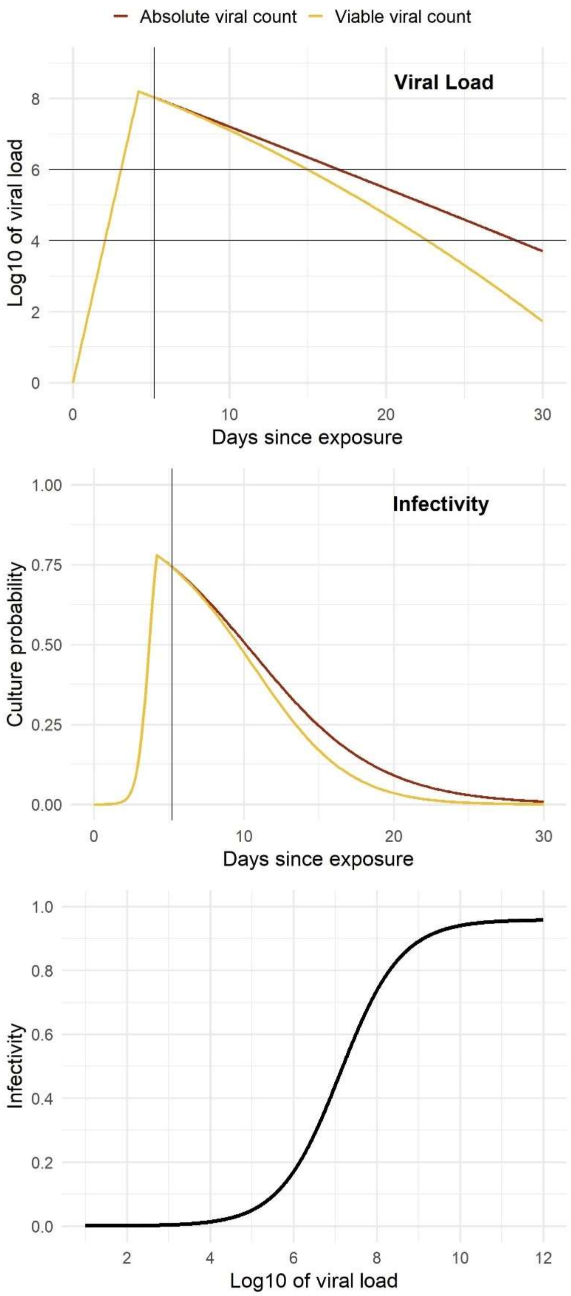

Figure S1. Infectivity as a function of the logarithm of the viral load, described by a sigmoidal curve (equation 3 in the Methods section). The vertical and horizontal lines represent, respectively, mean day of symptom onset and diagnostic limits of detection (for a given rapid test device, at $\mathbf{6}\ \boldsymbol{log_{10}}$ viral copies, and a PCR test at 4 $\boldsymbol{log_{10}}$ viral copies).

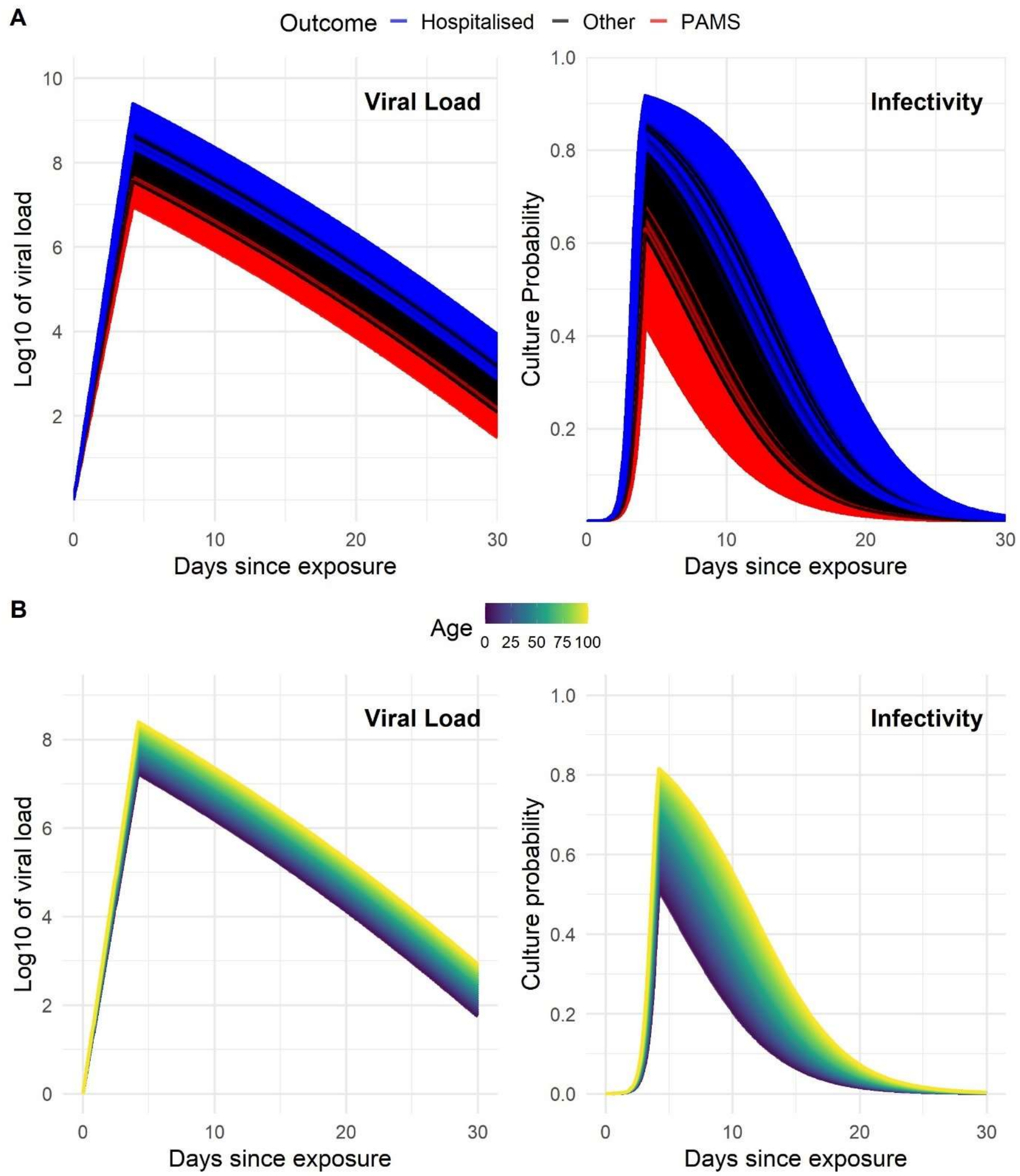

Figure S2. Simulated active viral load and infectivity profiles. Depicts 10,000 model generated viral load and infectivity profiles accounting for individual variation and the dependence between maximum viral load and clinical outcome (panel A) and age (panel B), as described in **equation (5)**. Model parameters used for these simulations are given in **Table S1**.

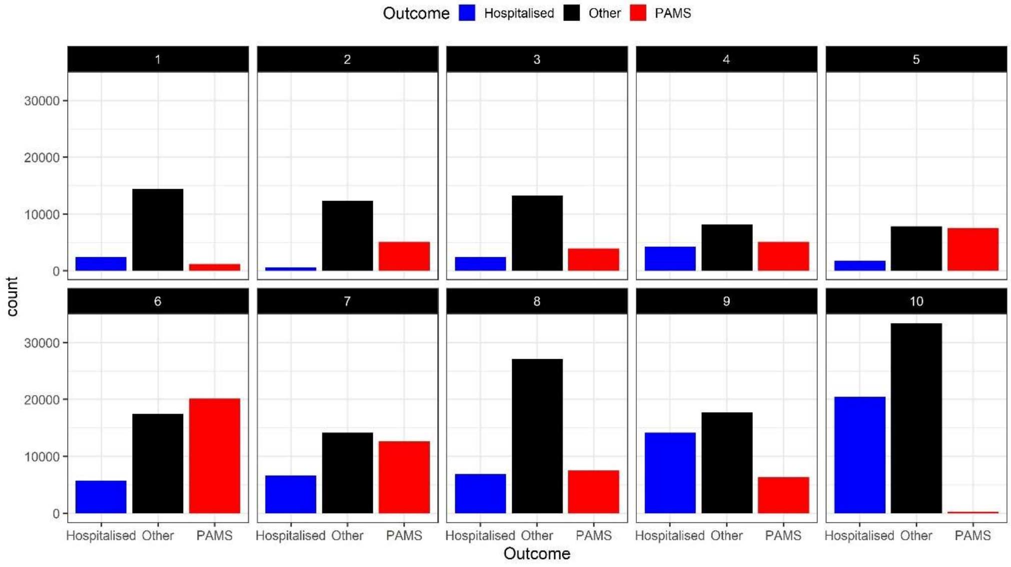

Figure S3. Distribution of clinical outcomes across age groups. Maps the frequency of each clinical outcome per modelled age groups (given by each panel). Age groups are coded as follows: 1) 0-4 yrs; 2) 5-9 yrs; 3) 10-14 yrs; 4) 15-19 yrs; 5) 20-24 yrs; 6) 25-34 yrs; 7) 35-44 yrs; 8) 45-55 yrs; 9) 55-64 yrs; 10) 65+ yrs.

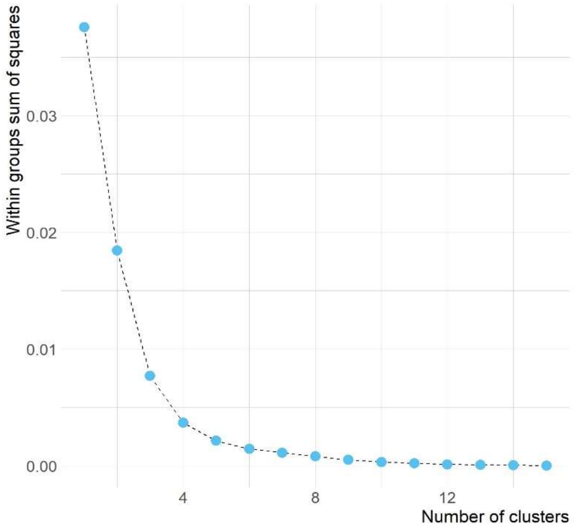

Figure S4. Testing strategy clustering. Depicts the result of the k-means clustering approach undertaken showing that the simulated testing strategies are most likely to cluster into 4 identifiable clusters.

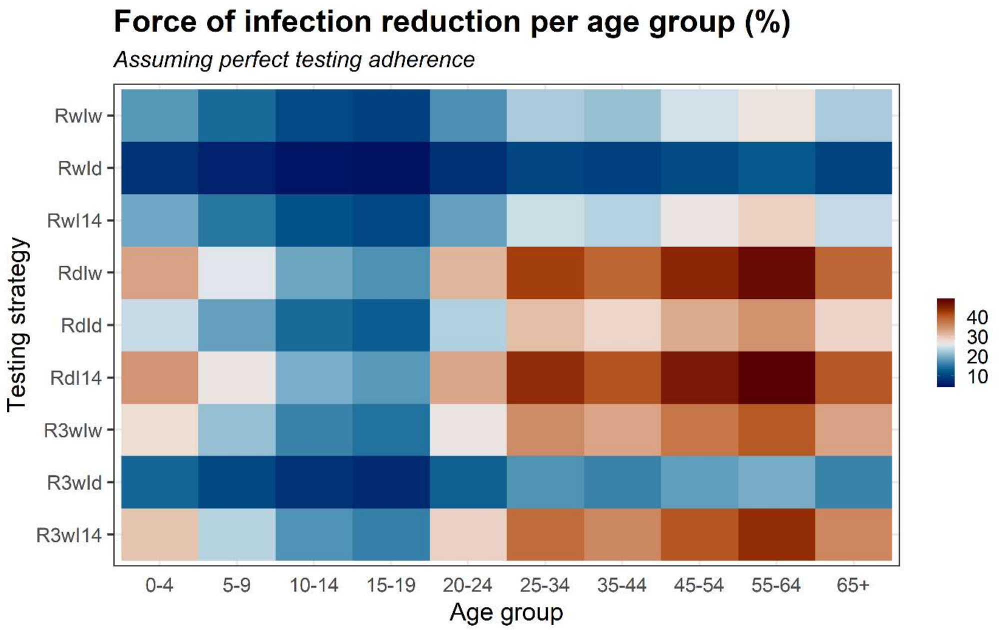

Figure S5. Expected reduction in force of infection for each of the strategies explored for Aim 1. This figure illustrates the total impact on the force of infection produced by each simulated testing strategy per age group. These estimates are calculated using equation (8) in the Methods section, while assuming perfect testing adherence.

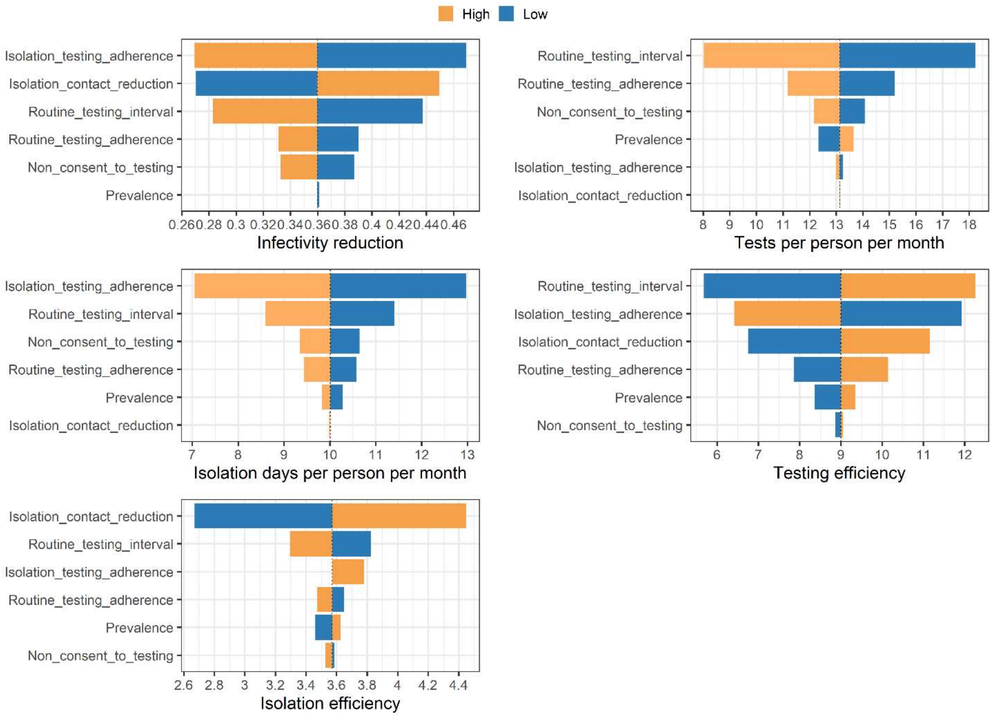

Figure S6. One-way sensitivity analysis of the Aim 2 simulation results. Each subplot illustrates the sensitivity of the respective model outcomes to the parameter inputs on the vertical axis. High, reference, and low values (respectively) are provided in Table 2.

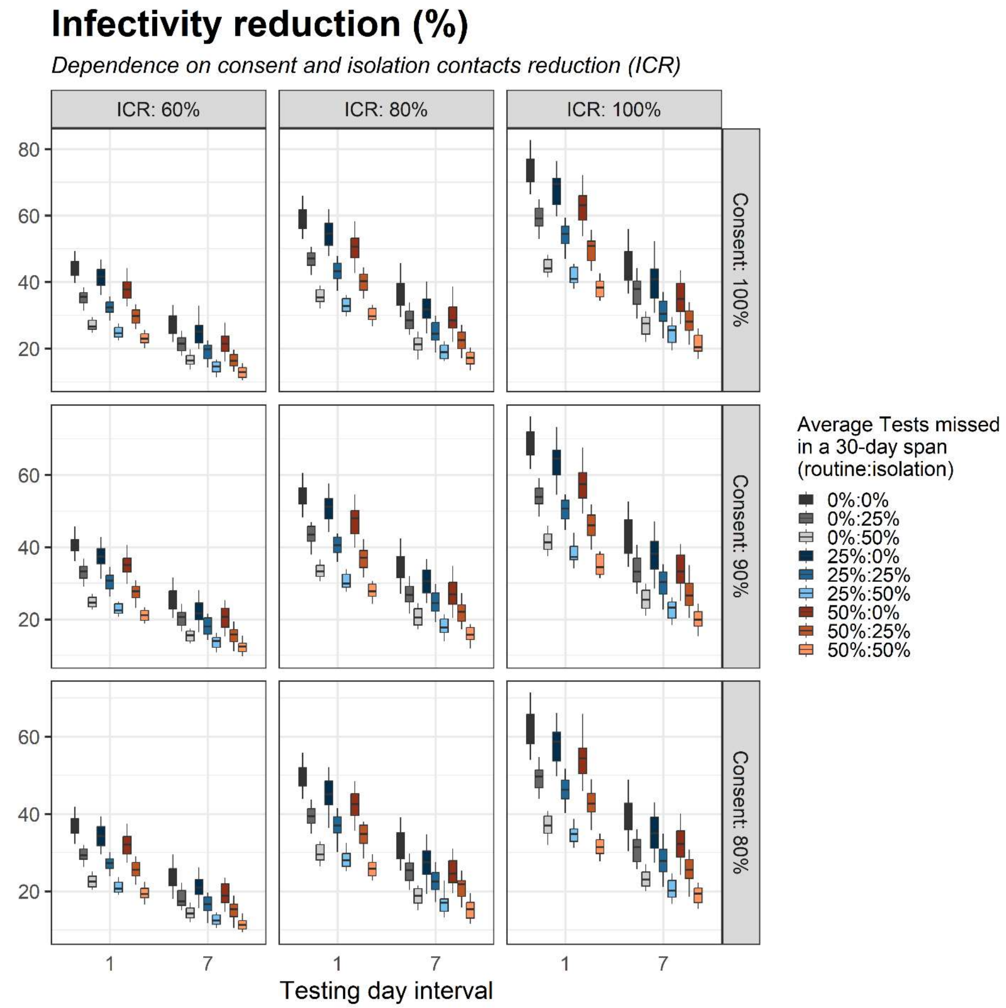

Figure S7. Aim 2 multi-way sensitivity analysis. Shows the expected infectivity reduction (y-axis) for strategies assuming all explored combinations of routine testing time interval (x-axis), testing adherence (colours), consent (horizontal panels) and reduction of contacts while isolating (vertical panels). The results shown here are aggregated across all isolation protocols.

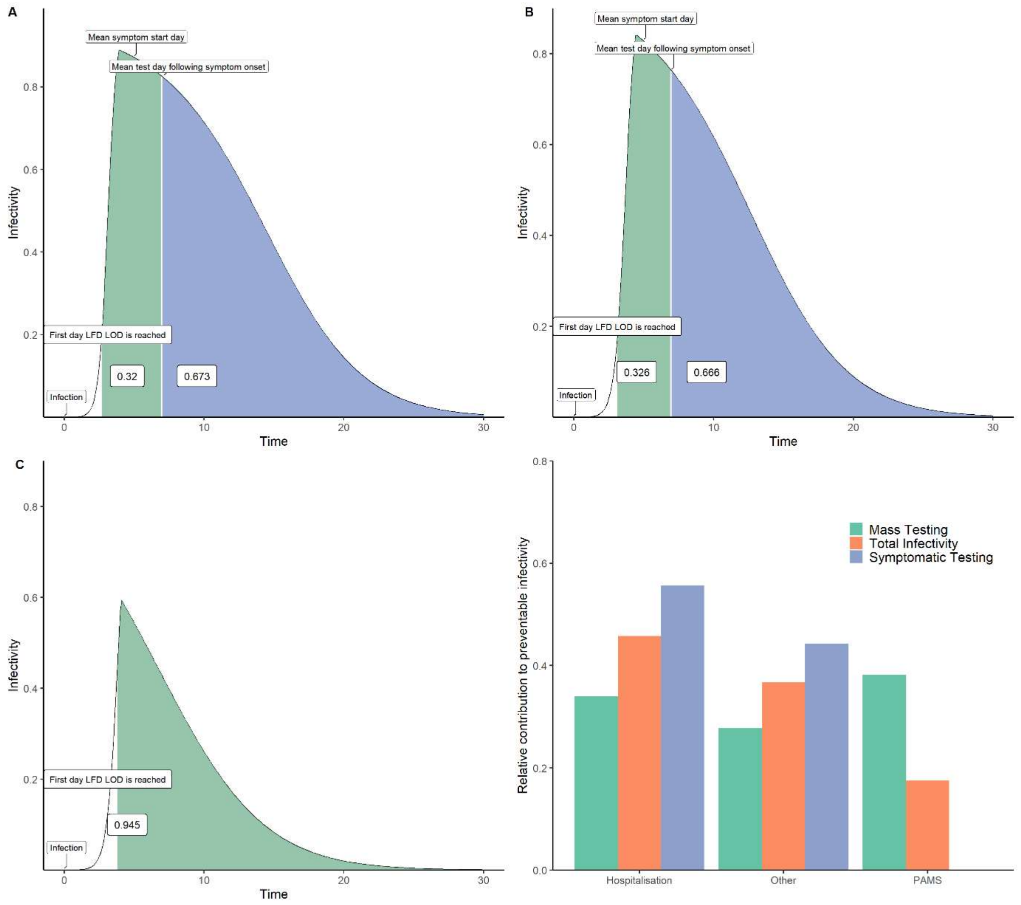

Figure S8. Hypothetical benefits of a routine testing strategy compared to testing symptomatic individuals only. Panels A-C show a typical infectivity profile for an individual who is hospitalised, has clinical symptoms or is asymptomatic respectively. The integral of the curve past the average day of testing following presentation of symptoms is coloured in blue. The time period where a routine asymptomatic test could result in a positive result is highlighted in green. Panel D presents the relative contribution to preventable infectivity (after the limit of detection has been reached) of each testing type, compared with the proportions of total infectivity per infection outcome. Routine testing, for example, would reduce the same amount of total preventable infectivity in hospitalised and asymptomatic patients.

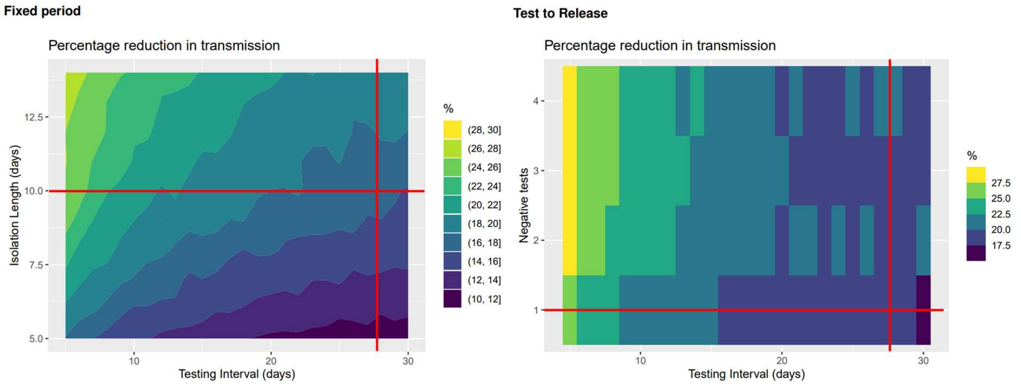

Figure S9. Model validation against Liverpool mass testing trial. Shows model outputs for regimens assuming either a fixed period of isolation (left) or a test to release protocol (right) for parameters consistent with the Liverpool Covid-SMART Community Testing Pilot. The red lines represent testing interval, isolation period and number of negative tests for release from isolation most consistent with the study.

Supplementary text

Simulation protocol

Briefly, the modelling framework presented here can be used by following the steps below:

1. Define a population of size N.
    a. Select population subgroups as required.
2. Assume a level of prevalence X.
    a. Calculate how many individuals are expected to be infected within 30 days.
    b. Allocate a day of infection to those infected individuals
3. Generate clinical outcomes for each individual (age-dependent).
    a. Generate a randomly distributed day of symptom onset for symptomatic individuals.
4. Generate viral load and infectivity profiles for each individual (age- and outcome-dependent) – **equation (5)**.
5. Specify a testing regimen.
6. Select the diagnostic tools to use (and in what proportions if using more than one).
    a. Calculate the expected number of false-positives Y given the testing frequency, prevalence, and test specificity. This enables the identification of Y random individuals who will mandatorily test positive and isolate within a 30-day period.
7. Implement the routine testing strategy
    a. Given the testing frequency, generate the day when each individual is tested within the 30-day window. Symptomatic individuals will always test one day after symptom onset.
    b. Given infected individual's viral load and test sensitivity determine which ones will test positive (true positives).
8. Implement the isolation protocol strategy
    a. For all those testing positive in 7b, determine when the positive test will be returned to them. Rapid diagnostic tests will have an instantaneous turnaround time, whereas PCR will have a predetermined fixed turnaround time, here set to 2 days.
    b. On receiving a positive test result individuals start isolating and a schedule for their test to release is determined. For each of those subsequent test timepoints, apply 7b until the isolation release criteria are met.
9. Output the infectivity over time, the number of tests performed, and the number of days spent isolating for each individual in the population.

Supplementary References

1 Jones, T. C. *et al*. Estimating infectiousness throughout SARS-CoV-2 infection course. *Science* **373** (2021). https://doi.org/10.1126/science.abi5273

2 Pickering, S. *et al*. Comparative performance of SARS-CoV-2 lateral flow antigen tests and association with detection of infectious virus in clinical specimens: a single-centre laboratory evaluation study. *Lancet Microbe* **2**, e461-e471 (2021). https://doi.org/10.1016/s2666-5247(21)00143-9

3 Department of Health and Social Care & UK Health Security Agency. *Performance of lateral flow devices during the COVID-19 pandemic*, <https://www.gov.uk/government/publications/lateral-flow-device-performance-data/performance-of-lateral-flow-devices-during-the-covid-19-pandemic#:~:text=LFDs%20were%20much%20better%20at,%2C%20the%20sensitivity%20was%2084.5%25.> (2022).

4 EY–Oxford Health Analytics Consortium. *Evaluation of the national COVID-19 testing programme in England between October 2020 and March 2022* (2023).
